# Syntheses and crystal structures of *catena*-poly[[di­iodido­zinc(II)]-μ-2,3-di­methyl­pyrazine-κ^2^*N*^1^:*N*^4^] and aqua­(2,3-di­methyl­pyrazine-κ*N*)di­iodidozinc(II)–2,3-di­methyl­pyrazine–water (2/1/1)

**DOI:** 10.1107/S2056989026001088

**Published:** 2026-02-03

**Authors:** Christian Näther, Gaurav Bhosekar

**Affiliations:** a Institut für Anorganische Chemie, Universität Kiel, Max-Eyth.-Str. 2, 24118 Kiel, Germany; b Suman Ramesh Tulsiani Technical Campus - Faculty of Engineering, Kamshet, Pune, India; University of Aberdeen, United Kingdom

**Keywords:** crystal structure, discrete complex, coordination polymer, synthesis, zinc iodide, 2,3-di­methyl­pyrazine

## Abstract

The reaction of zinc iodide with 2,3-di­methyl­pyrazine (C_6_H_8_N_2_) in ethanol leads to the formation of [ZnI_2_(C_6_H_8_N_2_)]_*n*_ (**1**), that according to powder X-ray diffraction was obtained as a pure phase. When the same reaction was performed in a mixture of ethanol and water as solvent, a few crystals of [ZnI_2_(C_6_H_8_N_2_)(H_2_O)]·0.5C_6_H_8_N_2_·0.5H_2_O (**2**) were serendipitiously obtained in a mixture with compound **1** as the major phase. The asymmetric unit of **1** consists of one zinc cation, two crystallographically independent iodide anions and one 2,3-di­methyl­pyrazine ligand all of them located in general positions. In the extended structure, the Zn cations are tetra­hedrally coordinated by two iodide anions and two symmetry-related 2,3-di­methyl­pyrazine ligands and are linked by bridging 2,3-di­methyl­pyrazine ligands into helical chains that proceed along the *c*-axis direction in the uncommon space group *P*3_2_. Within these chains, intra­chain C—H⋯I hydrogen bonding is observed. The asymmetric unit of **2** consists of two crystallographically independent [ZnI_2_(C_6_H_8_N_2_)(H_2_O)] complexes as well as one water mol­ecule and one none-coordinating 2,3-di­methyl­pyrazine ligand. In the complexes, the Zn cations are tetra­hedrally coordinated by two iodide anions, one 2,3-di­methyl­pyrazine ligand and one water mol­ecule. These complexes are packed in such a way that cavities are formed, which are filled by water and 2,3-di­methyl­pyrazine solvate mol­ecules that are hydrogen bonded to each other.

## Abstract 2

The syntheses and structures of [ZnI_2_(C_6_H_8_N_2_)]_*n*_ and [ZnI_2_(C_6_H_8_N_2_)(H_2_O)]·0.5C_6_H_8_N_2_·0.5H_2_O are reported (C_6_H_8_N_2_ = 2,3-di­methyl­pyrazine). In the first compound, the Zn^2+^ cations are connected by the 2,3-di­methyl­pyrazine ligands into helical chains, whereas in the second discrete complexes are observed that are arranged in such a way that cavities are formed, in which additional water and 2,3-di­methyl­pyrazine mol­ecules are incorporated.

## 1. Chemical context

Coordination compounds based on transition-metal halides and pseudo halides have been investigated for several decades because they show versatile structural behavior, which in part can be traced back to the fact that, in most cases, compounds of different stoichiometry are observed (Kromp & Sheldrick, 1999 ▸; Peng *et al.*, 2010 ▸). This is especially true for compounds based on Cu*X* (*X* = Cl, Br, I) that show typical Cu*X* substructures such as chains or layers (Li *et al.*, 2005 ▸; Näther *et al.*, 2001 ▸, 2002 ▸; Näther & Jess, 2002 ▸).

Compounds with chain-like metal–halide networks are also observed with Cd and Zn halides, even if the zinc compounds show a limited structural variability. However, in contrast to Cd^II^, where octa­hedral coordination is mostly observed, for Zn^II^ both tetra­hedral and octa­hedral coordination is found (Neumann *et al.*, 2018*a* ▸,*b* ▸). However, the Zn*X*_2_ and Cd*X*_2_ units can additionally be connected if bridging coligands are used, which is the case for example in compounds with pyrazine (C_4_H_4_N_2_), for which many examples are known (Bailey & Pennington, 1997 ▸,;Pickardt & Staub, 1997 ▸; Bhosekar *et al.*, 2006 ▸; Bourne *et al.*, 2001 ▸; Song *et al.*, 2004 ▸).

For such compounds with Zn halides, two different stoichiometries are observed that show a different ratio between the metal halide and the pyrazine coligand. They include the isotypic compounds [ZnCl_2_(C_4_H_4_N_2_)_2_] (Cambridge Structural Database refcode REMPAB; Bhosekar *et al.*, 2006 ▸) and [Br_2_(C_4_H_4_N_2_)_2_]_*n*_ (EBOLAI; Bourne *et al.*, 2001 ▸) in which the Zn cations are octa­hedrally coordinated and linked into layers by the pyrazine ligands. For the latter compound, a second modification of the same structure was also reported (EBOLAI01; Bhosekar *et al.*, 2006 ▸). The corresponding pyrazine-rich compound with ZnI_2_ is unknown.

In contrast, the pyrazine-deficient compounds of general composition [Zn*X*_2_(C_4_H_4_N_2_)] (*X* = Cl, Br, I) are all known. They include [ZnCl_2_(C_4_H_4_N_2_)] (TISTAQ; Pickardt & Staub, 1997 ▸) in which the Zn cations are connected into chains by pairs of μ-1,1-bridging halide anions that are further linked into layers by the pyrazine ligands. In contrast, [ZnBr_2_(C_4_H_4_N_2_)] (EBOKUB; Bourne *et al.*, 2001 ▸) and [ZnI_2_(C_4_H_4_N_2_)] [ISOPOV (Song *et al.*, 2004 ▸) and ISOPOV01 (Bhosekar *et al.*, 2006 ▸)] exhibit a different type of structure in which the Zn cations are tetra­hedrally coordinated and linked into corrugated chains by the pyrazine ligands.

In the course of our systematic work, we tried to prepare compounds based on 2,3-di­methyl­pyrazine (C_6_H_8_N_2_) to investigate the influence of the neutral coligand onto the structural behavior. Two compounds were prepared with ZnCl_2_, [ZnCl_2_(C_6_H_8_N_2_)] and [ZnCl_2_(C_6_H_8_N_2_)_2_] (Näther & Bhosekar, 2025*a* ▸). In both of these, the Zn cations are tetra­hedrally coordinated, leading to the formation of discrete complexes in the 2,3-di­methyl­pyrazine-rich compound, whereas in the 2,3-di­methyl­pyrazine-deficient compounds the Zn cations are linked into corrugated chains. Therefore, these structures are completely different from that with ZnCl_2_ and pyrazine. The 2,3-di­methyl­pyrazine-rich compound [ZnBr_2_(C_6_H_8_N_2_)_2_] is isotypic to [ZnCl_2_(C_6_H_8_N_2_)_2_] and con­sists of discrete complexes (Yang *et al.*, 2025 ▸), whereas in the 2,3-di­methyl­pyrazine-deficient compound [ZnBr_2_(C_6_H_8_N_2_)], the metal cations are linked into chains (Näther & Bhosekar, 2025*b* ▸).

Based on these results, we tried to prepare compounds starting from zinc iodide and 2,3-di­methyl­pyrazine to check if they show similar structures to those with ZnCl_2_ or ZnBr_2_. During the course of this work, the two title compounds were identified and characterized by single crystal X-ray diffraction.
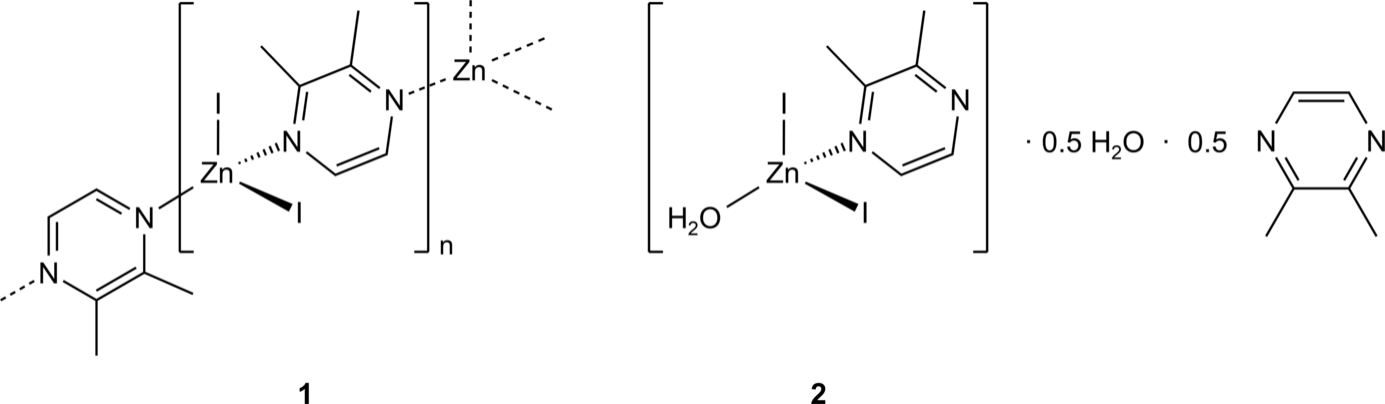


## 2. Structural commentary

**Figure 1:**
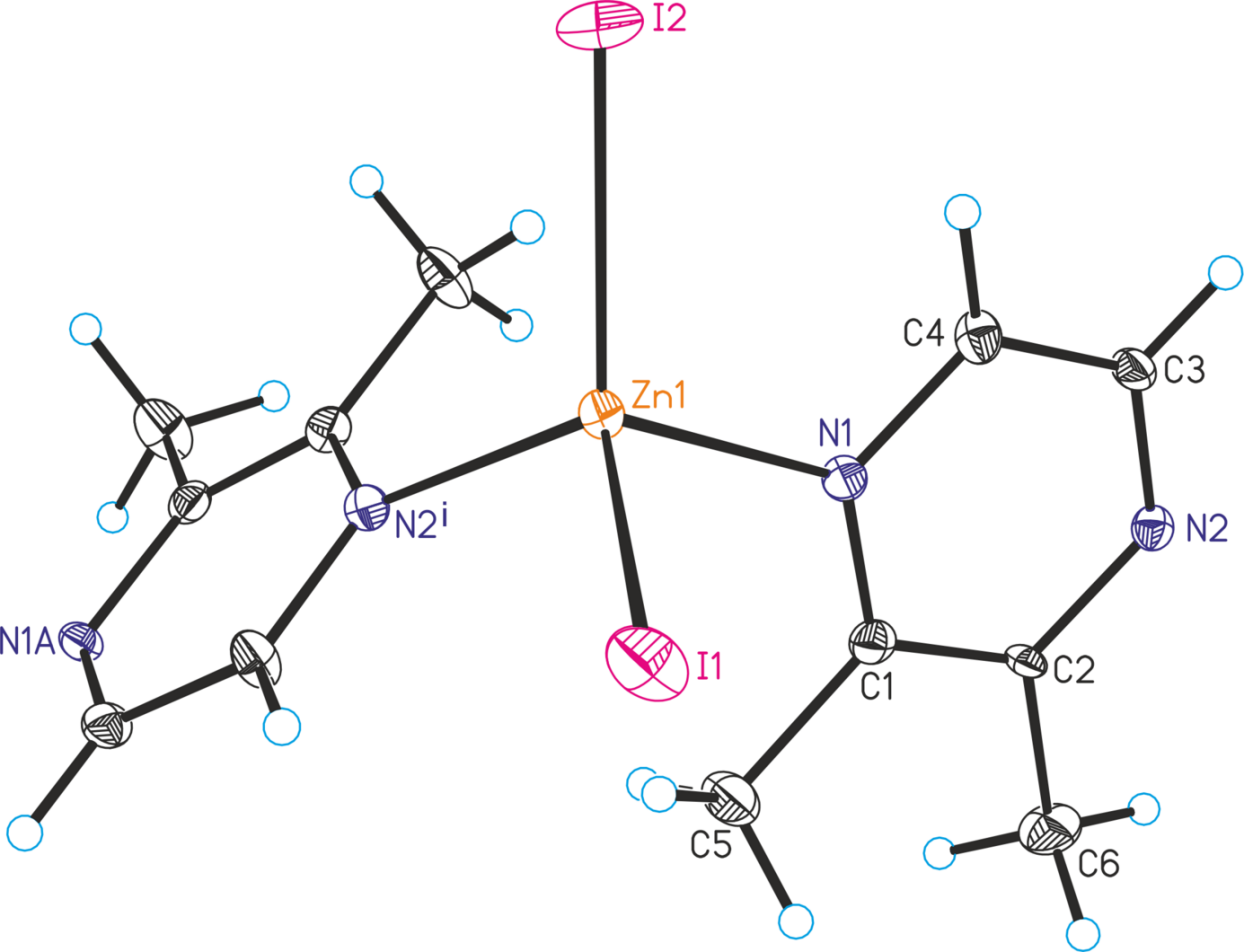
The asymmetric unit of **1** expanded to show the symmetry-generated bridging ligand with displacement ellipsoids drawn at the 50% probability level. Symmetry code: (i) −*y* + 1, *x* − *y* + 2, *z* − 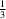.

**Figure 2:**
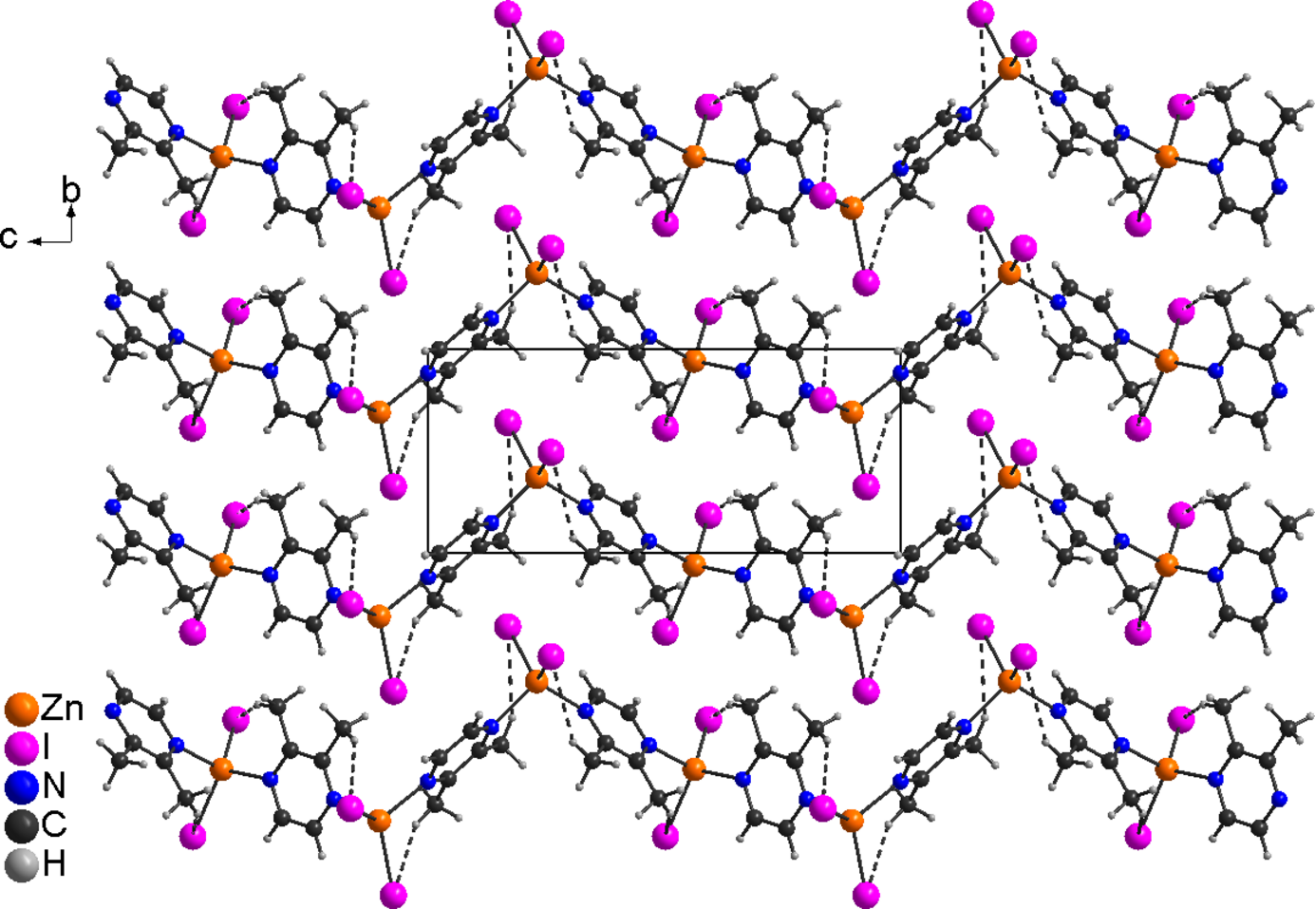
The crystal structure of **1** viewed along the crystallographic *a*-axis direction. Intra­chain C—H⋯I hydrogen bonds are shown as dashed lines.

The new compound [ZnI_2_(C_6_H_8_N_2_)] (**1**) is isotypic to the corresponding compounds [ZnCl_2_(C_6_H_8_N_2_)] and [ZnBr_2_(C_6_H_8_N_2_)] already reported in the literature (Näther & Bhosekar, 2025*a* ▸,*b* ▸). The asymmetric unit of compound **1** consists of one Zn cation, two crystallographically independent iodide anions and one 2,3-di­methyl­pyrazine ligand, all of them located in general positions (Fig. 1 ▸). In the extended structure the metal cations are tetra­hedrally coordinated by two N atoms of two symmetry-related 2,3-di­methyl­pyrazine ligands and two iodide anions. The spread of bond angles [102.9 (2)–114.97 (17)°] shows that the tetra­hedra are slightly distorted (Table 1 ▸). The Zn cations are linked into helical chains propagating along the crystallographic *c*-axis direction by the bridging 2,3-di­methyl­pyrazine ligands (Fig. 2 ▸). This structure is essentially the same as those of [ZnBr_2_(C_4_H_4_N_2_)] and [ZnI_2_(C_4_H_4_N_2_)] already reported in the literature (Bourne *et al.*, 2001 ▸; Song *et al.*, 2004 ▸; Bhosekar *et al.*, 2006 ▸).

**Figure 3:**
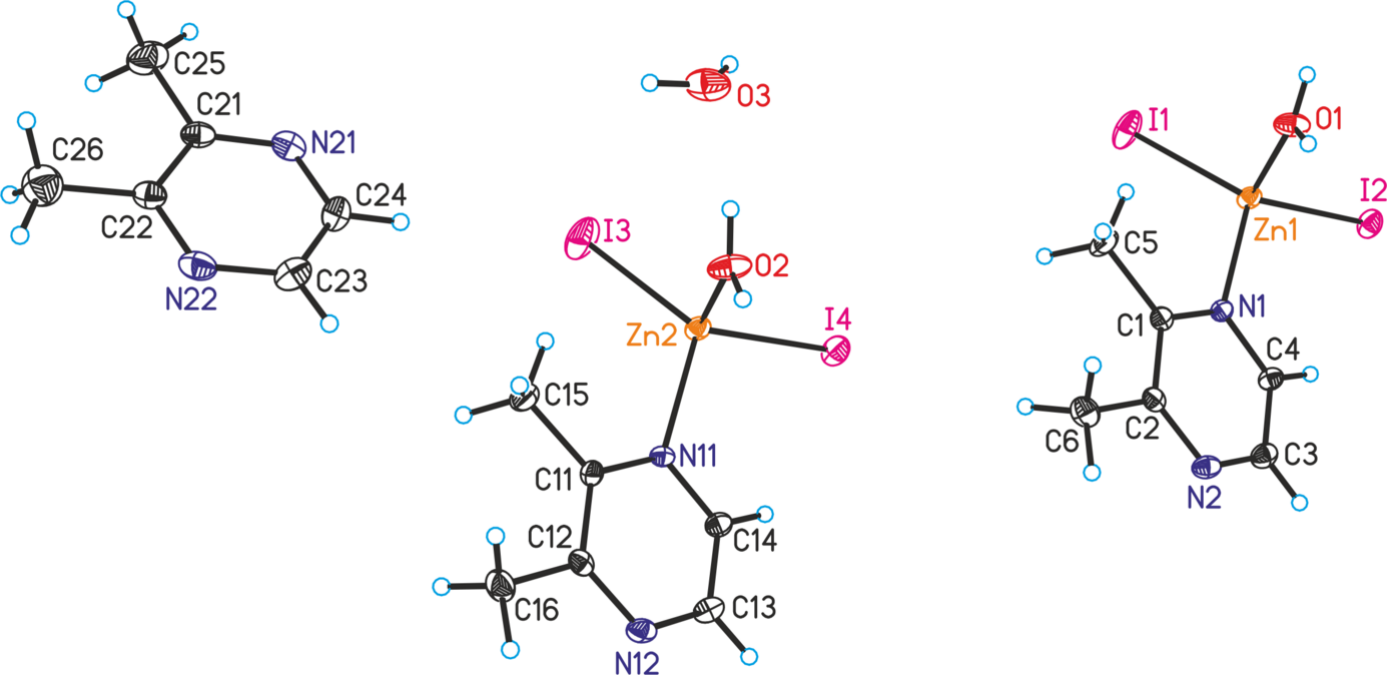
The asymmetric unit of **2** with displacement ellipsoids drawn at the 50% probability level.

The asymmetric unit of compound **2**, [ZnI_2_(C_6_H_8_N_2_)(H_2_O)]·0.5C_6_H_8_N_2_·0.5H_2_O, consists of two crystallographically independent Zn cations, four iodide anions as well as three 2,3-di­methyl­pyrazine ligands and three water mol­ecules, all of them located in general positions (Fig. 3 ▸). The Zn cations are tetra­hedrally coordinated by two iodide anions, one 2,3-di­methyl­pyrazine ligand and one water mol­ecule, forming discrete complexes (Fig. 3 ▸). Bond lengths and angles are very similar in both complexes (Table 2 ▸). It is noted, that no similar compound is reported with pyrazine and 2,3-di­methyl­pyrazine as well as Zn*X*_2_.

## 3. Supra­molecular features

In compound **1**, intra­chain C—H⋯I hydrogen bonds between the methyl H atoms and the iodide anions are observed (Table 3 ▸ and Fig. 2 ▸). There are additional C—H⋯I contacts between the chains, but the corresponding H⋯I distances and C—H⋯I angles indicate only weak inter­actions.

**Figure 4:**
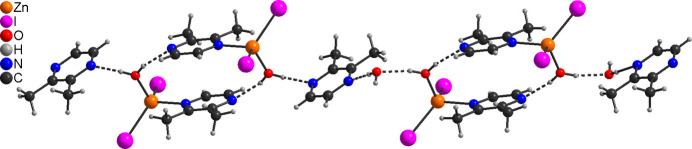
Fragment of the extended structure of **2** with view of a part of a [110] chain with O—H⋯N and O—H⋯O hydrogen bonds shown as dashed lines.

**Figure 5:**
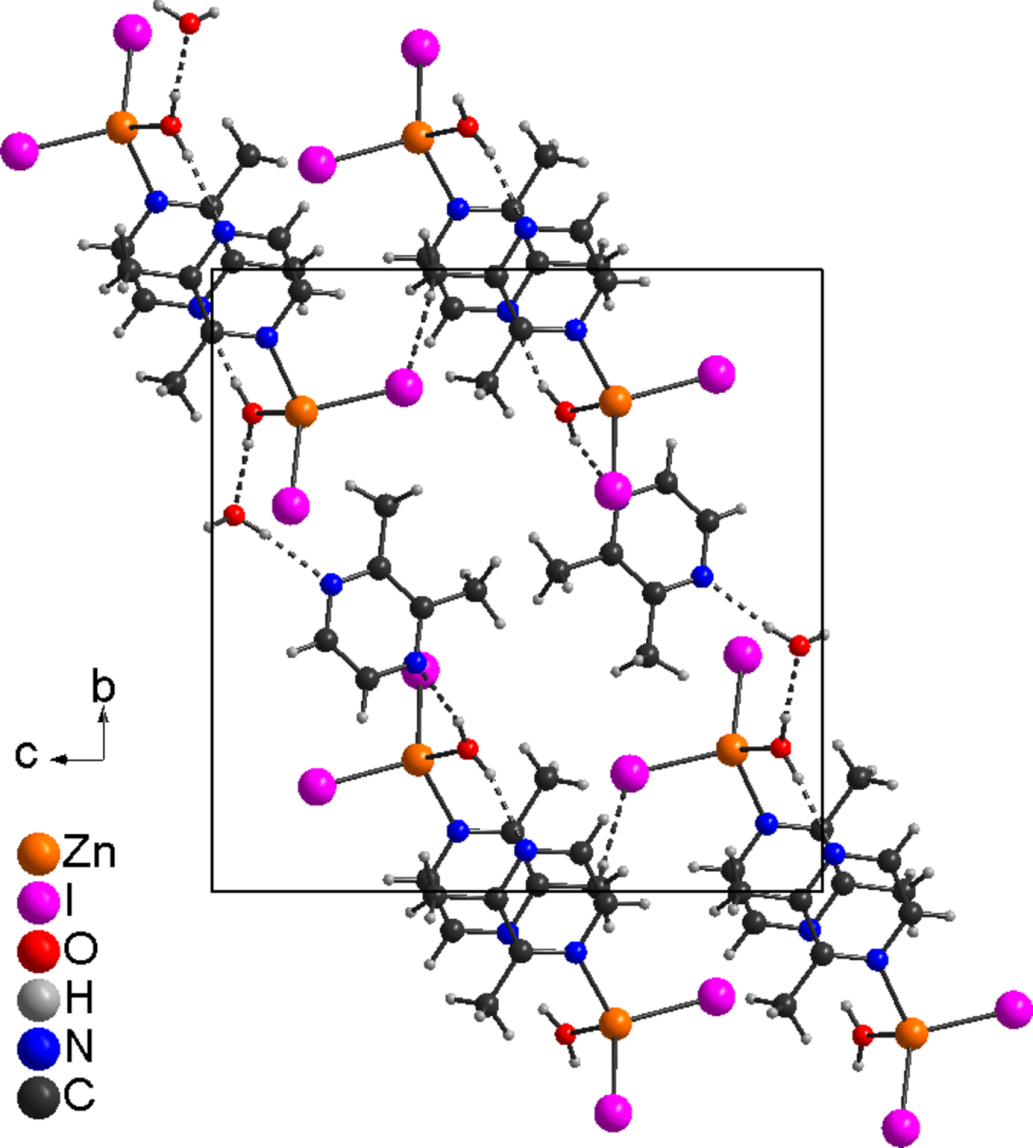
The crystal structure of **2** with view along the crystallographic *a*-axis direction. Hydrogen bonds are shown as dashed lines.

In compound **2**, two discrete complexes that are related by symmetry are linked into dimeric units by O—H⋯N hydrogen bonding between one of the H atoms of the coordinating water mol­ecules and 2,3-di­methyl­pyrazine ligands (Fig. 4 ▸ and Table 4 ▸). The second water H atom is hydrogen bonded to a further water mol­ecule that acts as acceptor and which is not involved in the metal coordination. This water mol­ecule make an O—H⋯N hydrogen bond to the uncoordinated 2,3-di­methyl­pyrazine ligand that is also connected to a further dimeric unit (Fig. 4 ▸). This means that the dimeric units are linked into chains by inter­molecular hydrogen bonding. Altogether two different dimeric units are observed, each of them are built up of one of the two crystallographically independent Zn complexes. The O—H⋯N and O—H⋯O hydrogen-bond angles are close to linear, indicating that these are strong inter­actions (Table 4 ▸). These chains are linked by additional hydrogen bonding, which also includes C—H⋯I inter­actions (Fig. 5 ▸ and Table 4 ▸).

## 4. Database survey

As already mentioned, compound **1** is isotypic to [ZnCl_2_(C_6_H_8_N_2_)] and [ZnBr_2_(C_6_H_8_N_2_)] already reported in the literature (Näther & Bhosekar, 2025*a* ▸,*b* ▸). Two additional 2,3-di­methyl­pyrazine-rich compounds with Zn halides of composition [ZnCl_2_(C_6_H_8_N_2_)_2_] (Näther & Bhosekar, 2025*a* ▸) and [ZnBr_2_(C_6_H_8_N_2_)_2_] (Yang *et al.*, 2025 ▸) have been reported that are isotypic and which form tetra­hedral discrete complexes. A search in the Cambridge Structural Database (Groom *et al.*, 2016 ▸, CSD Version 5.43, 2025) using CONQUEST (Bruno *et al.*, 2002 ▸) revealed that no further compounds containing divalent transition metal ions, halide ions and 2,3-di­methyl­pyrazine ligands have been reported.

Many more Zn^II^ compounds with halide ions and pyrazine are known and all of them are described in the *Structural commentary* above.

## 5. Synthesis and crystallization

**General**

Zinc iodide and 2,3-di­methyl­pyrazine were purchased from Sigma-Aldrich.

**Synthesis of 1**

0.25 mmol (79.8 mg) zinc iodide and 0.25 mmol (26.5 µL 2,3-di­methyl­pyrazine were reacted in 3 ml of ethanol. The reaction mixture was stirred for 2 d and the precipitate was filtered off and dried. Single crystals were obtained by using the same ratio of reactants without stirring.

**Figure 6:**
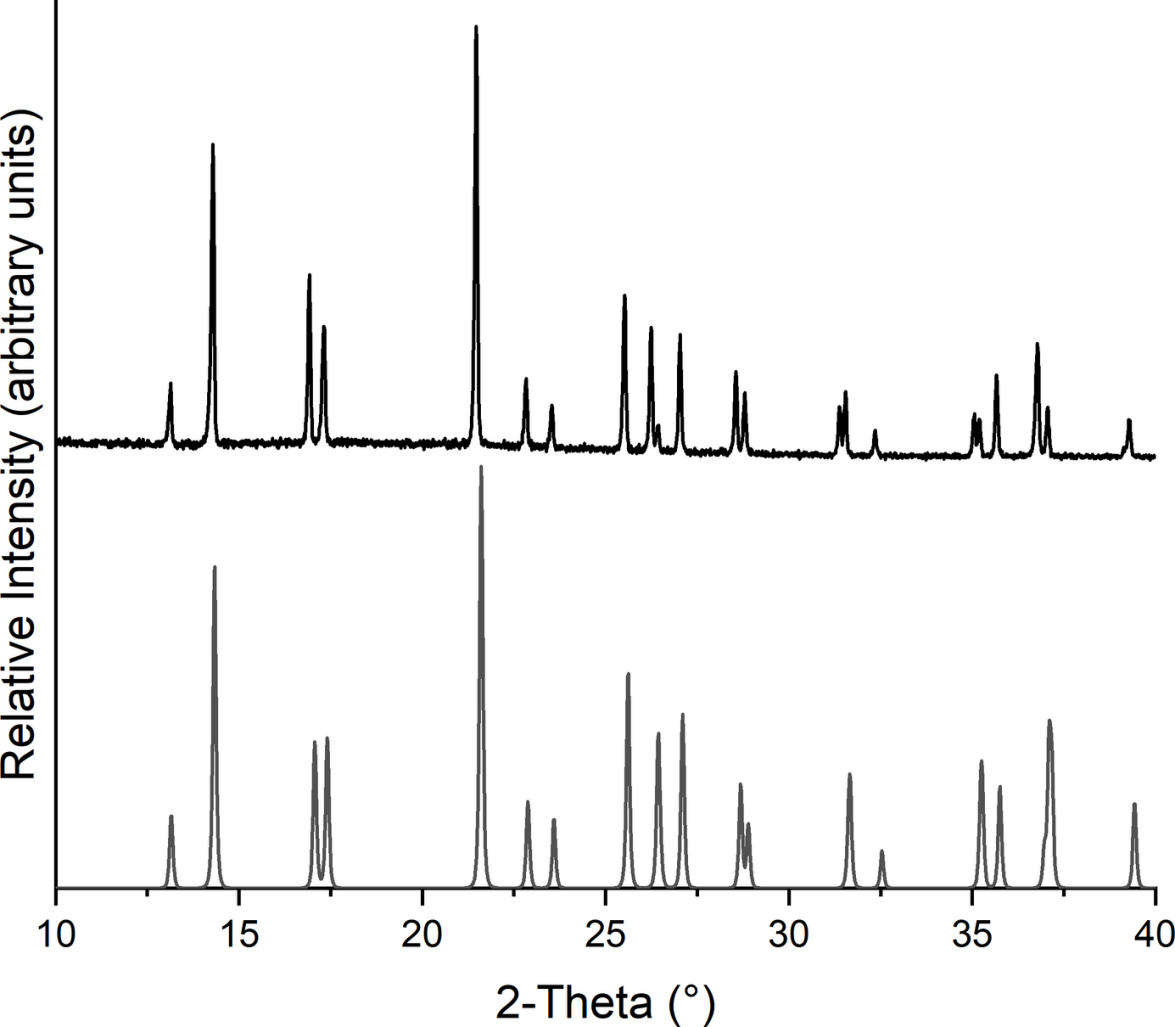
Experimental (top) and calculated (bottom) X-ray powder patterns of **1**.

Compound **1** was additionally investigated by X-ray powder diffraction and the experimental pattern was compared with that calculated from single crystal data. This reveals that a pure sample has been obtained (Fig. 6 ▸).

**Synthesis of 2**

A few crystals were accidentally obtained by the reaction of 0.25 mmol (79.8 mg) zinc iodide and 0.25 mmol (26.5 µl 2,3-di­methyl­pyrazine in 3 ml of a mixture (1:1) of ethanol and water). This batch consisted predominantly of **1** as the major phase, with traces of **2** as the minor phase.

**Experimental details**

The PXRD measurements were performed with a Stoe Transmission Powder Diffraction System (STADI P) with Cu *K*α_1_ radiation (λ = 1.540598 Å) equipped with a MYTHEN 1K detector and a Johansson-type Ge(111) monochromator.

## 6. Refinement

Crystal data, data collection and structure refinement details are summarized in Table 5 ▸. The C—H hydrogen atoms were positioned with idealized geometry (methyl H atoms allowed to rotate but not to tip) and were refined isotropically with *U*_iso_(H) = 1.2*U*_eq_(C) (1.5 for methyl H atoms). The O—H hydrogen atoms in **2** were located in a difference map, their bond lengths were set to ideal values and finally they were refined isotropically with *U*_iso_(H) = 1.5*U*_eq_(O).

## Supplementary Material

Crystal structure: contains datablock(s) 1, 2. DOI: 10.1107/S2056989026001088/hb8191sup1.cif

Structure factors: contains datablock(s) 1. DOI: 10.1107/S2056989026001088/hb81911sup2.hkl

Structure factors: contains datablock(s) 2. DOI: 10.1107/S2056989026001088/hb81912sup3.hkl

CCDC references: 2527702, 2527701

Additional supporting information:  crystallographic information; 3D view; checkCIF report

## Figures and Tables

**Table 1:** Selected geometric parameters (Å, °) for **1**[Chem scheme1]

Zn1—N2^i^	2.098 (6)	Zn1—I1	2.5374 (10)
Zn1—N1	2.117 (6)	Zn1—I2	2.5454 (9)
			
N2^i^—Zn1—N1	102.9 (2)	N2^i^—Zn1—I2	114.97 (17)
N2^i^—Zn1—I1	110.53 (17)	N1—Zn1—I2	109.77 (17)
N1—Zn1—I1	104.68 (17)	I1—Zn1—I2	113.01 (4)

Symmetry code: (i) 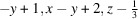.

**Table 2:** Selected geometric parameters (Å, °) for **2**[Chem scheme1]

Zn1—O1	2.002 (3)	Zn2—O2	1.983 (3)
Zn1—N1	2.087 (3)	Zn2—N11	2.108 (3)
Zn1—I1	2.5290 (4)	Zn2—I3	2.5427 (4)
Zn1—I2	2.5671 (4)	Zn2—I4	2.5578 (4)
			
O1—Zn1—N1	98.85 (10)	O2—Zn2—N11	97.91 (10)
O1—Zn1—I1	109.69 (7)	O2—Zn2—I3	108.60 (8)
N1—Zn1—I1	115.81 (8)	N11—Zn2—I3	118.84 (7)
O1—Zn1—I2	109.52 (8)	O2—Zn2—I4	109.55 (8)
N1—Zn1—I2	109.17 (7)	N11—Zn2—I4	106.59 (7)
I1—Zn1—I2	112.837 (15)	I3—Zn2—I4	113.988 (16)

**Table 3:** Hydrogen-bond geometry (Å, °) for **1**[Chem scheme1]

*D*—H⋯*A*	*D*—H	H⋯*A*	*D*⋯*A*	*D*—H⋯*A*
C3—H3⋯I1^ii^	0.95	3.07	3.767 (7)	132
C3—H3⋯I2^iii^	0.95	3.15	3.844 (7)	131
C4—H4⋯I2	0.95	3.07	3.763 (7)	131
C5—H5*C*⋯I1	0.98	3.12	3.987 (9)	148
C6—H6*A*⋯I2^ii^	0.98	3.10	3.977 (9)	150
C6—H6*C*⋯I1^iv^	0.98	3.23	3.924 (8)	130

Symmetry codes: (ii) 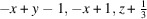; (iii) 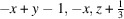; (iv) 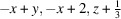.

**Table 4:** Hydrogen-bond geometry (Å, °) for **2**[Chem scheme1]

*D*—H⋯*A*	*D*—H	H⋯*A*	*D*⋯*A*	*D*—H⋯*A*
O1—H1*O*1⋯N2^i^	0.84	2.00	2.798 (4)	159
O1—H2*O*1⋯N22^ii^	0.84	1.94	2.758 (4)	164
C3—H3⋯I2^iii^	0.95	3.17	4.050 (3)	154
C4—H4⋯I1^iii^	0.95	3.32	4.003 (3)	131
C4—H4⋯I2	0.95	3.06	3.754 (3)	131
C5—H5*A*⋯I1	0.98	3.13	3.900 (4)	137
C6—H6*B*⋯I4	0.98	3.24	4.204 (4)	169
C6—H6*C*⋯I1^iv^	0.98	3.15	4.076 (4)	157
O2—H1*O*2⋯N12^v^	0.84	2.00	2.830 (4)	169
O2—H2*O*2⋯O3	0.84	1.83	2.652 (4)	168
C13—H13⋯I4^vi^	0.95	3.09	3.954 (3)	151
C14—H14⋯I4	0.95	2.97	3.669 (3)	131
C15—H15*B*⋯I2^vii^	0.98	3.29	4.236 (4)	162
C15—H15*C*⋯I3	0.98	3.08	3.967 (4)	151
C16—H16*B*⋯I2^vii^	0.98	3.11	4.060 (4)	163
O3—H1*O*3⋯N21^viii^	0.84	1.97	2.810 (4)	174
O3—H2*O*3⋯I4^iv^	0.84	3.00	3.486 (3)	119
C25—H25*B*⋯I2^ix^	0.98	3.30	4.277 (4)	173
C26—H26*A*⋯I2^x^	0.98	3.22	4.109 (4)	151
C26—H26*C*⋯I1^viii^	0.98	3.27	3.987 (4)	132

Symmetry codes: (i) 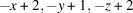; (ii) 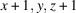; (iii) 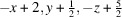; (iv) 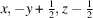; (v) 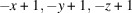; (vi) 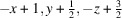; (vii) 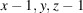; (viii) 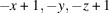; (ix) 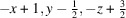; (x) 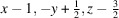.

**Table 5:** Experimental details

	**1**	**2**
Crystal data		
Chemical formula	[ZnI_2_(C_6_H_8_N_2_)]	[ZnI_2_(C_6_H_8_N_2_)(H_2_O)]·0.5C_6_H_8_N_2._0.5H_2_O
*M*_r_	427.31	508.41
Crystal system, space group	Trigonal, *P*3_2_	Monoclinic, *P*2_1_/*c*
Temperature (K)	170	170
*a*, *b*, *c* (Å)	7.7674 (4), 7.7674 (4), 15.5731 (10)	14.4026 (8), 14.5960 (8), 14.5524 (8)
α, β, γ (°)	90, 90, 120	90, 100.262 (7), 90
*V* (Å^3^)	813.69 (10)	3010.3 (3)
*Z*	3	8
Radiation type	Mo *K*α	Mo *K*α
μ (mm^−1^)	7.90	5.73
Crystal size (mm)	0.15 × 0.10 × 0.06	0.2 × 0.15 × 0.12

Data collection		
Diffractometer	Stoe IPDS1	Stoe IPDS2
Absorption correction	Numerical (*X-RED* and *X-SHAPE*; Stoe, 2008 ▸)	Numerical (*X-RED* and *X-SHAPE*; Stoe, 2008 ▸)
*T*_min_, *T*_max_	0.276, 0.410	0.363, 0.540
No. of measured, independent and observed [*I* > 2σ(*I*)] reflections	4792, 2537, 2392	25407, 7267, 6098
*R*_int_	0.039	0.042
(sin θ/λ)_max_ (Å^−1^)	0.661	0.662

Refinement		
*R*[*F*^2^ > 2σ(*F*^2^)], *wR*(*F*^2^), *S*	0.026, 0.061, 0.99	0.029, 0.073, 1.08
No. of reflections	2537	7267
No. of parameters	103	300
No. of restraints	1	0
H-atom treatment	H-atom parameters constrained	H-atom parameters constrained
Δρ_max_, Δρ_min_ (e Å^−3^)	0.63, −0.85	1.00, −1.27
Absolute structure	Flack *x* determined using 1115 quotients [(*I*^+^)−(*I*^−^)]/[(*I*^+^)+(*I*^−^)] (Parsons *et al.*, 2013 ▸)	–
Absolute structure parameter	0.02 (3)	–

Computer programs: *X-AREA* (Stoe, 2008 ▸), *SHELXT* (Sheldrick, 2015*a* ▸), *SHELXL* (Sheldrick, 2015*b* ▸), *DIAMOND* (Brandenburg, 1999 ▸), *XP* in *SHELXTL-PC* (Sheldrick, 2008 ▸) and *publCIF* (Westrip, 2010 ▸).

## supplementary crystallographic information

### catena-Poly[[diiodidozinc(II)]-µ-2,3-dimethylpyrazine-κ2N1:N4] (1) Crystal data

**Table:**

[ZnI_2_(C_6_H_8_N_2_)]	*D*_x_ = 2.616 Mg m^−^^3^
*M**_r_* = 427.31	Mo *K*α radiation, λ = 0.71073 Å
Trigonal, *P*3_2_	Cell parameters from 5827 reflections
*a* = 7.7674 (4) Å	θ = 3.0–28.0°
*c* = 15.5731 (10) Å	µ = 7.90 mm^−^^1^
*V* = 813.69 (10) Å^3^	*T* = 170 K
*Z* = 3	Block, light yellow
*F*(000) = 582	0.15 × 0.10 × 0.06 mm

### catena-Poly[[diiodidozinc(II)]-µ-2,3-dimethylpyrazine-κ2N1:N4] (1) Data collection

**Table:**

Stoe IPDS-1 diffractometer	2392 reflections with *I* > 2σ(*I*)
phi scans	*R*_int_ = 0.039
Absorption correction: numerical (X-Red and X-Shape; Stoe, 2008)	θ_max_ = 28.0°, θ_min_ = 3.0°
*T*_min_ = 0.276, *T*_max_ = 0.410	*h* = −9→10
4792 measured reflections	*k* = −10→10
2537 independent reflections	*l* = −19→20

### catena-Poly[[diiodidozinc(II)]-µ-2,3-dimethylpyrazine-κ2N1:N4] (1) Refinement

**Table:**

Refinement on *F*^2^	H-atom parameters constrained
Least-squares matrix: full	*w* = 1/[σ^2^(*F*_o_^2^) + (0.0384*P*)^2^] where *P* = (*F*_o_^2^ + 2*F*_c_^2^)/3
*R*[*F*^2^ > 2σ(*F*^2^)] = 0.026	(Δ/σ)_max_ < 0.001
*wR*(*F*^2^) = 0.061	Δρ_max_ = 0.63 e Å^−^^3^
*S* = 0.99	Δρ_min_ = −0.85 e Å^−^^3^
2537 reflections	Extinction correction: SHELXL-2016/6 (Sheldrick 2015b), Fc^*^=kFc[1+0.001xFc^2^λ^3^/sin(2θ)]^-1/4^
103 parameters	Extinction coefficient: 0.0103 (9)
1 restraint	Absolute structure: Flack *x* determined using 1115 quotients [(*I*^+^)-(*I*^-^)]/[(*I*^+^)+(*I*^-^)] (Parsons *et al.*, 2013)
Hydrogen site location: inferred from neighbouring sites	Absolute structure parameter: 0.02 (3)

### catena-Poly[[diiodidozinc(II)]-µ-2,3-dimethylpyrazine-κ2N1:N4] (1) Special details

**Table:**

Geometry. All esds (except the esd in the dihedral angle between two l.s. planes) are estimated using the full covariance matrix. The cell esds are taken into account individually in the estimation of esds in distances, angles and torsion angles; correlations between esds in cell parameters are only used when they are defined by crystal symmetry. An approximate (isotropic) treatment of cell esds is used for estimating esds involving l.s. planes.

### catena-Poly[[diiodidozinc(II)]-µ-2,3-dimethylpyrazine-κ2N1:N4] (1) Fractional atomic coordinates and isotropic or equivalent isotropic displacement parameters (Å^2^)

**Table:**

	*x*	*y*	*z*	*U*_iso_*/*U*_eq_	
Zn1	0.05935 (12)	0.68899 (12)	0.10408 (6)	0.01099 (19)	
I1	0.38766 (7)	0.74884 (8)	0.16515 (4)	0.02133 (16)	
I2	−0.18375 (8)	0.32372 (7)	0.07388 (4)	0.02393 (16)	
N1	−0.0583 (9)	0.7944 (9)	0.1999 (4)	0.0101 (11)	
C1	0.0185 (10)	0.9843 (10)	0.2250 (5)	0.0112 (14)	
C2	−0.0696 (10)	1.0353 (10)	0.2916 (5)	0.0103 (13)	
N2	−0.2263 (9)	0.8921 (9)	0.3342 (4)	0.0105 (12)	
C3	−0.2994 (11)	0.7034 (11)	0.3101 (5)	0.0129 (14)	
H3	−0.409720	0.601457	0.340202	0.015*	
C4	−0.2185 (11)	0.6525 (11)	0.2422 (5)	0.0119 (14)	
H4	−0.275917	0.517318	0.225335	0.014*	
C5	0.2000 (12)	1.1410 (12)	0.1802 (6)	0.0197 (16)	
H5A	0.284006	1.244768	0.221352	0.030*	
H5B	0.160336	1.199538	0.133956	0.030*	
H5C	0.274449	1.081275	0.155958	0.030*	
C6	0.0168 (13)	1.2473 (12)	0.3193 (6)	0.0201 (17)	
H6A	−0.073296	1.258001	0.360166	0.030*	
H6B	0.034368	1.330469	0.269001	0.030*	
H6C	0.145925	1.292253	0.346720	0.030*	

### catena-Poly[[diiodidozinc(II)]-µ-2,3-dimethylpyrazine-κ2N1:N4] (1) Atomic displacement parameters (Å^2^)

**Table:**

	*U*^11^	*U*^22^	*U*^33^	*U*^12^	*U*^13^	*U*^23^
Zn1	0.0114 (4)	0.0107 (4)	0.0099 (4)	0.0048 (3)	0.0010 (3)	0.0008 (3)
I1	0.0135 (2)	0.0245 (3)	0.0252 (3)	0.0090 (2)	−0.0019 (2)	0.0083 (2)
I2	0.0226 (3)	0.0122 (2)	0.0299 (3)	0.0034 (2)	−0.0046 (2)	−0.0066 (2)
N1	0.012 (3)	0.008 (3)	0.011 (3)	0.006 (2)	0.001 (2)	0.000 (2)
C1	0.012 (3)	0.011 (3)	0.010 (4)	0.005 (3)	−0.003 (3)	0.001 (3)
C2	0.009 (3)	0.007 (3)	0.011 (4)	0.000 (3)	0.000 (3)	0.002 (3)
N2	0.011 (3)	0.011 (3)	0.010 (3)	0.006 (2)	0.000 (2)	0.000 (2)
C3	0.013 (3)	0.010 (3)	0.012 (4)	0.002 (3)	0.005 (3)	−0.001 (3)
C4	0.014 (3)	0.012 (3)	0.012 (4)	0.008 (3)	0.002 (3)	0.001 (3)
C5	0.018 (4)	0.014 (3)	0.021 (4)	0.004 (3)	0.008 (3)	−0.001 (3)
C6	0.023 (4)	0.013 (4)	0.022 (5)	0.007 (3)	0.000 (3)	−0.007 (3)

### catena-Poly[[diiodidozinc(II)]-µ-2,3-dimethylpyrazine-κ2N1:N4] (1) Geometric parameters (Å, º)

**Table:**

Zn1—N2^i^	2.098 (6)	N2—C3	1.334 (9)
Zn1—N1	2.117 (6)	C3—C4	1.384 (10)
Zn1—I1	2.5374 (10)	C3—H3	0.9500
Zn1—I2	2.5454 (9)	C4—H4	0.9500
N1—C1	1.343 (9)	C5—H5A	0.9800
N1—C4	1.351 (10)	C5—H5B	0.9800
C1—C2	1.405 (11)	C5—H5C	0.9800
C1—C5	1.496 (10)	C6—H6A	0.9800
C2—N2	1.343 (9)	C6—H6B	0.9800
C2—C6	1.497 (10)	C6—H6C	0.9800
			
N2^i^—Zn1—N1	102.9 (2)	N2—C3—C4	121.6 (7)
N2^i^—Zn1—I1	110.53 (17)	N2—C3—H3	119.2
N1—Zn1—I1	104.68 (17)	C4—C3—H3	119.2
N2^i^—Zn1—I2	114.97 (17)	N1—C4—C3	120.3 (7)
N1—Zn1—I2	109.77 (17)	N1—C4—H4	119.9
I1—Zn1—I2	113.01 (4)	C3—C4—H4	119.9
C1—N1—C4	118.4 (6)	C1—C5—H5A	109.5
C1—N1—Zn1	126.1 (5)	C1—C5—H5B	109.5
C4—N1—Zn1	115.4 (5)	H5A—C5—H5B	109.5
N1—C1—C2	120.8 (6)	C1—C5—H5C	109.5
N1—C1—C5	118.6 (7)	H5A—C5—H5C	109.5
C2—C1—C5	120.6 (6)	H5B—C5—H5C	109.5
N2—C2—C1	119.9 (6)	C2—C6—H6A	109.5
N2—C2—C6	119.4 (7)	C2—C6—H6B	109.5
C1—C2—C6	120.6 (6)	H6A—C6—H6B	109.5
C3—N2—C2	118.9 (7)	C2—C6—H6C	109.5
C3—N2—Zn1^ii^	116.2 (5)	H6A—C6—H6C	109.5
C2—N2—Zn1^ii^	125.0 (5)	H6B—C6—H6C	109.5

Symmetry codes: (i) −*y*+1, *x*−*y*+2, *z*−1/3; (ii) −*x*+*y*−1, −*x*+1, *z*+1/3.

### catena-Poly[[diiodidozinc(II)]-µ-2,3-dimethylpyrazine-κ2N1:N4] (1) Hydrogen-bond geometry (Å, º)

**Table:**

*D*—H···*A*	*D*—H	H···*A*	*D*···*A*	*D*—H···*A*
C3—H3···I1^ii^	0.95	3.07	3.767 (7)	132
C3—H3···I2^iii^	0.95	3.15	3.844 (7)	131
C4—H4···I2	0.95	3.07	3.763 (7)	131
C5—H5*C*···I1	0.98	3.12	3.987 (9)	148
C6—H6*A*···I2^ii^	0.98	3.10	3.977 (9)	150
C6—H6*C*···I1^iv^	0.98	3.23	3.924 (8)	130

Symmetry codes: (ii) −*x*+*y*−1, −*x*+1, *z*+1/3; (iii) −*x*+*y*−1, −*x*, *z*+1/3; (iv) −*x*+*y*, −*x*+2, *z*+1/3.

### Aqua(2,3-dimethylpyrazine-κN)diiodidozinc(II)–2,3-dimethylpyrazine–water (2/1/1) (2) Crystal data

**Table:**

[ZnI_2_(C_6_H_8_N_2_)(H_2_O)]·0.5C_6_H_8_N_2._0.5H_2_O	*F*(000) = 1904
*M**_r_* = 508.41	*D*_x_ = 2.244 Mg m^−^^3^
Monoclinic, *P*2_1_/*c*	Mo *K*α radiation, λ = 0.71073 Å
*a* = 14.4026 (8) Å	Cell parameters from 8000 reflections
*b* = 14.5960 (8) Å	θ = 13.7–25.1°
*c* = 14.5524 (8) Å	µ = 5.73 mm^−^^1^
β = 100.262 (7)°	*T* = 170 K
*V* = 3010.3 (3) Å^3^	Block, light yellow
*Z* = 8	0.2 × 0.15 × 0.12 mm

### Aqua(2,3-dimethylpyrazine-κN)diiodidozinc(II)–2,3-dimethylpyrazine–water (2/1/1) (2) Data collection

**Table:**

Stoe IPDS-2 diffractometer	6098 reflections with *I* > 2σ(*I*)
ω scans	*R*_int_ = 0.042
Absorption correction: numerical (X-Red and X-Shape; Stoe, 2008)	θ_max_ = 28.1°, θ_min_ = 2.3°
*T*_min_ = 0.363, *T*_max_ = 0.540	*h* = −19→18
25407 measured reflections	*k* = −19→19
7267 independent reflections	*l* = −19→18

### Aqua(2,3-dimethylpyrazine-κN)diiodidozinc(II)–2,3-dimethylpyrazine–water (2/1/1) (2) Refinement

**Table:**

Refinement on *F*^2^	Hydrogen site location: mixed
Least-squares matrix: full	H-atom parameters constrained
*R*[*F*^2^ > 2σ(*F*^2^)] = 0.029	*w* = 1/[σ^2^(*F*_o_^2^) + (0.0426*P*)^2^] where *P* = (*F*_o_^2^ + 2*F*_c_^2^)/3
*wR*(*F*^2^) = 0.073	(Δ/σ)_max_ = 0.003
*S* = 1.08	Δρ_max_ = 1.00 e Å^−^^3^
7267 reflections	Δρ_min_ = −1.27 e Å^−^^3^
300 parameters	Extinction correction: SHELXL-2016/6 (Sheldrick 2015b), Fc^*^=kFc[1+0.001xFc^2^λ^3^/sin(2θ)]^-1/4^
0 restraints	Extinction coefficient: 0.00133 (8)

### Aqua(2,3-dimethylpyrazine-κN)diiodidozinc(II)–2,3-dimethylpyrazine–water (2/1/1) (2) Special details

**Table:**

Geometry. All esds (except the esd in the dihedral angle between two l.s. planes) are estimated using the full covariance matrix. The cell esds are taken into account individually in the estimation of esds in distances, angles and torsion angles; correlations between esds in cell parameters are only used when they are defined by crystal symmetry. An approximate (isotropic) treatment of cell esds is used for estimating esds involving l.s. planes.

### Aqua(2,3-dimethylpyrazine-κN)diiodidozinc(II)–2,3-dimethylpyrazine–water (2/1/1) (2) Fractional atomic coordinates and isotropic or equivalent isotropic displacement parameters (Å^2^)

**Table:**

	*x*	*y*	*z*	*U*_iso_*/*U*_eq_	
Zn1	1.00802 (3)	0.28769 (2)	1.16169 (2)	0.01361 (9)	
I1	0.90891 (2)	0.14426 (2)	1.15794 (2)	0.02788 (8)	
I2	1.08961 (2)	0.33138 (2)	1.32735 (2)	0.01948 (7)	
O1	1.10521 (19)	0.27087 (16)	1.08038 (17)	0.0203 (5)	
H1O1	1.119909	0.310939	1.044087	0.030*	
H2O1	1.141729	0.226419	1.095758	0.030*	
N1	0.93938 (19)	0.40255 (18)	1.09643 (18)	0.0126 (5)	
C1	0.8953 (2)	0.4044 (2)	1.0068 (2)	0.0128 (6)	
C2	0.8609 (2)	0.4888 (2)	0.9660 (2)	0.0129 (6)	
N2	0.8710 (2)	0.56644 (18)	1.01447 (19)	0.0152 (5)	
C3	0.9127 (2)	0.5622 (2)	1.1056 (2)	0.0161 (6)	
H3	0.918518	0.616452	1.142228	0.019*	
C4	0.9466 (2)	0.4815 (2)	1.1457 (2)	0.0131 (6)	
H4	0.975773	0.480775	1.209605	0.016*	
C5	0.8845 (3)	0.3180 (2)	0.9515 (2)	0.0205 (7)	
H5A	0.922482	0.269643	0.986639	0.031*	
H5B	0.905872	0.327979	0.892014	0.031*	
H5C	0.817976	0.299679	0.939448	0.031*	
C6	0.8126 (3)	0.4930 (2)	0.8661 (2)	0.0196 (7)	
H6A	0.798324	0.556914	0.848475	0.029*	
H6B	0.753868	0.457679	0.858311	0.029*	
H6C	0.854104	0.467107	0.826234	0.029*	
Zn2	0.50774 (3)	0.27165 (2)	0.64683 (2)	0.01409 (9)	
I3	0.41962 (2)	0.11991 (2)	0.62960 (2)	0.03425 (8)	
I4	0.58366 (2)	0.31016 (2)	0.81492 (2)	0.02073 (7)	
O2	0.6061 (2)	0.26861 (17)	0.56732 (18)	0.0236 (6)	
H1O2	0.619096	0.316302	0.539850	0.035*	
H2O2	0.635716	0.220472	0.559460	0.035*	
N11	0.43695 (19)	0.39088 (18)	0.59044 (18)	0.0118 (5)	
C11	0.3943 (2)	0.3999 (2)	0.5007 (2)	0.0132 (6)	
C12	0.3637 (2)	0.4869 (2)	0.4651 (2)	0.0130 (6)	
N12	0.3745 (2)	0.56096 (19)	0.5200 (2)	0.0157 (5)	
C13	0.4137 (2)	0.5495 (2)	0.6105 (2)	0.0170 (6)	
H13	0.419765	0.600984	0.651137	0.020*	
C14	0.4451 (2)	0.4658 (2)	0.6456 (2)	0.0145 (6)	
H14	0.473065	0.460451	0.709600	0.017*	
C15	0.3816 (3)	0.3172 (2)	0.4392 (3)	0.0241 (8)	
H15A	0.411429	0.327849	0.384576	0.036*	
H15B	0.314157	0.305456	0.418564	0.036*	
H15C	0.411051	0.264128	0.474000	0.036*	
C16	0.3182 (3)	0.4990 (2)	0.3652 (2)	0.0206 (7)	
H16A	0.312766	0.564493	0.350465	0.031*	
H16B	0.255257	0.471264	0.354917	0.031*	
H16C	0.356851	0.469191	0.324871	0.031*	
O3	0.6746 (2)	0.10498 (19)	0.53661 (18)	0.0302 (6)	
H1O3	0.686717	0.075199	0.586713	0.045*	
H2O3	0.629747	0.086549	0.495824	0.045*	
N21	0.2873 (2)	0.0063 (2)	0.3036 (2)	0.0215 (6)	
C21	0.2315 (3)	−0.0204 (2)	0.2254 (2)	0.0184 (7)	
C22	0.1958 (3)	0.0433 (2)	0.1553 (2)	0.0188 (7)	
N22	0.2185 (2)	0.1320 (2)	0.1662 (2)	0.0235 (6)	
C23	0.2765 (3)	0.1577 (3)	0.2449 (3)	0.0257 (8)	
H23	0.294382	0.220238	0.253393	0.031*	
C24	0.3102 (3)	0.0953 (3)	0.3129 (2)	0.0235 (7)	
H24	0.350639	0.115537	0.368033	0.028*	
C25	0.2079 (3)	−0.1205 (3)	0.2143 (3)	0.0315 (9)	
H25A	0.236644	−0.146040	0.163742	0.047*	
H25B	0.139247	−0.127966	0.199178	0.047*	
H25C	0.232357	−0.152645	0.272677	0.047*	
C26	0.1311 (3)	0.0153 (3)	0.0680 (3)	0.0341 (9)*	
H26A	0.115193	0.069040	0.027845	0.051*	
H26B	0.073315	−0.010799	0.083847	0.051*	
H26C	0.162397	−0.030555	0.034942	0.051*	

### Aqua(2,3-dimethylpyrazine-κN)diiodidozinc(II)–2,3-dimethylpyrazine–water (2/1/1) (2) Atomic displacement parameters (Å^2^)

**Table:**

	*U*^11^	*U*^22^	*U*^33^	*U*^12^	*U*^13^	*U*^23^
Zn1	0.0179 (2)	0.00929 (17)	0.01323 (17)	0.00008 (14)	0.00160 (13)	0.00240 (12)
I1	0.03984 (15)	0.01969 (12)	0.02286 (12)	−0.01600 (11)	0.00216 (10)	0.00215 (9)
I2	0.02425 (13)	0.01724 (11)	0.01470 (11)	−0.00273 (9)	−0.00265 (8)	0.00094 (7)
O1	0.0264 (13)	0.0130 (11)	0.0236 (12)	0.0055 (10)	0.0106 (10)	0.0074 (9)
N1	0.0132 (13)	0.0102 (12)	0.0144 (12)	−0.0008 (10)	0.0023 (10)	0.0010 (9)
C1	0.0123 (14)	0.0130 (14)	0.0130 (13)	−0.0003 (12)	0.0022 (11)	0.0012 (11)
C2	0.0101 (14)	0.0122 (14)	0.0167 (15)	−0.0013 (12)	0.0032 (11)	0.0046 (11)
N2	0.0187 (14)	0.0111 (12)	0.0170 (13)	0.0010 (11)	0.0061 (10)	0.0028 (10)
C3	0.0177 (16)	0.0131 (15)	0.0189 (15)	−0.0021 (13)	0.0069 (12)	0.0006 (12)
C4	0.0172 (16)	0.0123 (14)	0.0099 (13)	0.0004 (12)	0.0029 (11)	−0.0008 (11)
C5	0.0291 (19)	0.0118 (15)	0.0182 (16)	−0.0007 (14)	−0.0020 (13)	−0.0041 (12)
C6	0.0204 (17)	0.0220 (17)	0.0150 (15)	0.0022 (14)	−0.0006 (13)	0.0042 (12)
Zn2	0.0182 (2)	0.00990 (17)	0.01384 (17)	−0.00048 (14)	0.00192 (14)	0.00160 (13)
I3	0.04443 (17)	0.02050 (13)	0.03637 (15)	−0.01783 (12)	0.00329 (12)	0.00158 (10)
I4	0.02988 (13)	0.01643 (11)	0.01352 (11)	−0.00158 (9)	−0.00256 (8)	0.00195 (7)
O2	0.0353 (15)	0.0123 (11)	0.0277 (13)	0.0040 (11)	0.0174 (11)	0.0046 (9)
N11	0.0128 (12)	0.0097 (12)	0.0131 (12)	0.0013 (10)	0.0030 (9)	0.0013 (9)
C11	0.0117 (14)	0.0126 (14)	0.0152 (14)	−0.0020 (12)	0.0018 (11)	−0.0010 (11)
C12	0.0089 (14)	0.0146 (15)	0.0160 (15)	−0.0009 (12)	0.0035 (11)	0.0027 (11)
N12	0.0152 (14)	0.0110 (13)	0.0219 (14)	0.0000 (11)	0.0059 (11)	0.0039 (10)
C13	0.0215 (17)	0.0127 (14)	0.0179 (15)	−0.0008 (13)	0.0067 (12)	−0.0014 (12)
C14	0.0163 (16)	0.0142 (15)	0.0137 (14)	0.0008 (13)	0.0047 (11)	−0.0019 (11)
C15	0.032 (2)	0.0161 (16)	0.0206 (17)	0.0040 (15)	−0.0061 (14)	−0.0053 (13)
C16	0.0201 (17)	0.0201 (17)	0.0196 (16)	−0.0001 (14)	−0.0020 (13)	0.0049 (12)
O3	0.0434 (17)	0.0255 (14)	0.0220 (13)	0.0123 (13)	0.0064 (12)	0.0076 (10)
N21	0.0205 (15)	0.0208 (15)	0.0235 (15)	0.0036 (13)	0.0046 (12)	0.0037 (11)
C21	0.0181 (17)	0.0174 (16)	0.0219 (16)	0.0034 (13)	0.0093 (13)	0.0005 (12)
C22	0.0202 (17)	0.0183 (16)	0.0191 (16)	0.0035 (14)	0.0062 (13)	0.0012 (13)
N22	0.0266 (16)	0.0187 (14)	0.0251 (15)	0.0066 (13)	0.0044 (12)	0.0050 (12)
C23	0.031 (2)	0.0187 (17)	0.0279 (18)	−0.0020 (16)	0.0067 (15)	−0.0029 (14)
C24	0.0250 (18)	0.0246 (18)	0.0196 (16)	−0.0017 (16)	0.0005 (13)	−0.0048 (13)
C25	0.031 (2)	0.0188 (18)	0.047 (2)	−0.0041 (17)	0.0110 (17)	−0.0029 (16)

### Aqua(2,3-dimethylpyrazine-κN)diiodidozinc(II)–2,3-dimethylpyrazine–water (2/1/1) (2) Geometric parameters (Å, º)

**Table:**

Zn1—O1	2.002 (3)	C11—C15	1.494 (4)
Zn1—N1	2.087 (3)	C12—N12	1.337 (4)
Zn1—I1	2.5290 (4)	C12—C16	1.494 (4)
Zn1—I2	2.5671 (4)	N12—C13	1.348 (4)
O1—H1O1	0.8400	C13—C14	1.369 (5)
O1—H2O1	0.8401	C13—H13	0.9500
N1—C1	1.345 (4)	C14—H14	0.9500
N1—C4	1.351 (4)	C15—H15A	0.9800
C1—C2	1.418 (4)	C15—H15B	0.9800
C1—C5	1.488 (4)	C15—H15C	0.9800
C2—N2	1.329 (4)	C16—H16A	0.9800
C2—C6	1.496 (4)	C16—H16B	0.9800
N2—C3	1.356 (4)	C16—H16C	0.9800
C3—C4	1.366 (4)	O3—H1O3	0.8400
C3—H3	0.9500	O3—H2O3	0.8400
C4—H4	0.9500	N21—C21	1.328 (5)
C5—H5A	0.9800	N21—C24	1.342 (5)
C5—H5B	0.9800	C21—C22	1.408 (5)
C5—H5C	0.9800	C21—C25	1.502 (5)
C6—H6A	0.9800	C22—N22	1.337 (5)
C6—H6B	0.9800	C22—C26	1.492 (5)
C6—H6C	0.9800	N22—C23	1.346 (5)
Zn2—O2	1.983 (3)	C23—C24	1.369 (5)
Zn2—N11	2.108 (3)	C23—H23	0.9500
Zn2—I3	2.5427 (4)	C24—H24	0.9500
Zn2—I4	2.5578 (4)	C25—H25A	0.9800
O2—H1O2	0.8401	C25—H25B	0.9800
O2—H2O2	0.8400	C25—H25C	0.9800
N11—C11	1.348 (4)	C26—H26A	0.9800
N11—C14	1.349 (4)	C26—H26B	0.9800
C11—C12	1.412 (4)	C26—H26C	0.9800
			
O1—Zn1—N1	98.85 (10)	C12—C11—C15	120.7 (3)
O1—Zn1—I1	109.69 (7)	N12—C12—C11	120.8 (3)
N1—Zn1—I1	115.81 (8)	N12—C12—C16	118.0 (3)
O1—Zn1—I2	109.52 (8)	C11—C12—C16	121.2 (3)
N1—Zn1—I2	109.17 (7)	C12—N12—C13	117.9 (3)
I1—Zn1—I2	112.837 (15)	N12—C13—C14	121.8 (3)
Zn1—O1—H1O1	124.6	N12—C13—H13	119.1
Zn1—O1—H2O1	114.1	C14—C13—H13	119.1
H1O1—O1—H2O1	119.3	N11—C14—C13	120.9 (3)
C1—N1—C4	118.4 (3)	N11—C14—H14	119.5
C1—N1—Zn1	124.1 (2)	C13—C14—H14	119.5
C4—N1—Zn1	117.3 (2)	C11—C15—H15A	109.5
N1—C1—C2	119.5 (3)	C11—C15—H15B	109.5
N1—C1—C5	119.6 (3)	H15A—C15—H15B	109.5
C2—C1—C5	120.9 (3)	C11—C15—H15C	109.5
N2—C2—C1	121.4 (3)	H15A—C15—H15C	109.5
N2—C2—C6	117.9 (3)	H15B—C15—H15C	109.5
C1—C2—C6	120.6 (3)	C12—C16—H16A	109.5
C2—N2—C3	117.9 (3)	C12—C16—H16B	109.5
N2—C3—C4	121.3 (3)	H16A—C16—H16B	109.5
N2—C3—H3	119.4	C12—C16—H16C	109.5
C4—C3—H3	119.4	H16A—C16—H16C	109.5
N1—C4—C3	121.4 (3)	H16B—C16—H16C	109.5
N1—C4—H4	119.3	H1O3—O3—H2O3	117.5
C3—C4—H4	119.3	C21—N21—C24	118.1 (3)
C1—C5—H5A	109.5	N21—C21—C22	121.0 (3)
C1—C5—H5B	109.5	N21—C21—C25	117.9 (3)
H5A—C5—H5B	109.5	C22—C21—C25	121.1 (3)
C1—C5—H5C	109.5	N22—C22—C21	120.2 (3)
H5A—C5—H5C	109.5	N22—C22—C26	117.9 (3)
H5B—C5—H5C	109.5	C21—C22—C26	121.9 (3)
C2—C6—H6A	109.5	C22—N22—C23	118.2 (3)
C2—C6—H6B	109.5	N22—C23—C24	121.1 (3)
H6A—C6—H6B	109.5	N22—C23—H23	119.5
C2—C6—H6C	109.5	C24—C23—H23	119.5
H6A—C6—H6C	109.5	N21—C24—C23	121.4 (3)
H6B—C6—H6C	109.5	N21—C24—H24	119.3
O2—Zn2—N11	97.91 (10)	C23—C24—H24	119.3
O2—Zn2—I3	108.60 (8)	C21—C25—H25A	109.5
N11—Zn2—I3	118.84 (7)	C21—C25—H25B	109.5
O2—Zn2—I4	109.55 (8)	H25A—C25—H25B	109.5
N11—Zn2—I4	106.59 (7)	C21—C25—H25C	109.5
I3—Zn2—I4	113.988 (16)	H25A—C25—H25C	109.5
Zn2—O2—H1O2	120.1	H25B—C25—H25C	109.5
Zn2—O2—H2O2	122.0	C22—C26—H26A	109.5
H1O2—O2—H2O2	117.9	C22—C26—H26B	109.5
C11—N11—C14	118.3 (3)	H26A—C26—H26B	109.5
C11—N11—Zn2	124.3 (2)	C22—C26—H26C	109.5
C14—N11—Zn2	116.9 (2)	H26A—C26—H26C	109.5
N11—C11—C12	120.1 (3)	H26B—C26—H26C	109.5
N11—C11—C15	119.2 (3)		

### Aqua(2,3-dimethylpyrazine-κN)diiodidozinc(II)–2,3-dimethylpyrazine–water (2/1/1) (2) Hydrogen-bond geometry (Å, º)

**Table:**

*D*—H···*A*	*D*—H	H···*A*	*D*···*A*	*D*—H···*A*
O1—H1*O*1···N2^i^	0.84	2.00	2.798 (4)	159
O1—H2*O*1···N22^ii^	0.84	1.94	2.758 (4)	164
C3—H3···I2^iii^	0.95	3.17	4.050 (3)	154
C4—H4···I1^iii^	0.95	3.32	4.003 (3)	131
C4—H4···I2	0.95	3.06	3.754 (3)	131
C5—H5*A*···I1	0.98	3.13	3.900 (4)	137
C6—H6*B*···I4	0.98	3.24	4.204 (4)	169
C6—H6*C*···I1^iv^	0.98	3.15	4.076 (4)	157
O2—H1*O*2···N12^v^	0.84	2.00	2.830 (4)	169
O2—H2*O*2···O3	0.84	1.83	2.652 (4)	168
C13—H13···I4^vi^	0.95	3.09	3.954 (3)	151
C14—H14···I4	0.95	2.97	3.669 (3)	131
C15—H15*B*···I2^vii^	0.98	3.29	4.236 (4)	162
C15—H15*C*···I3	0.98	3.08	3.967 (4)	151
C16—H16*B*···I2^vii^	0.98	3.11	4.060 (4)	163
O3—H1*O*3···N21^viii^	0.84	1.97	2.810 (4)	174
O3—H2*O*3···I4^iv^	0.84	3.00	3.486 (3)	119
C25—H25*B*···I2^ix^	0.98	3.30	4.277 (4)	173
C26—H26*A*···I2^x^	0.98	3.22	4.109 (4)	151
C26—H26*C*···I1^viii^	0.98	3.27	3.987 (4)	132

Symmetry codes: (i) −*x*+2, −*y*+1, −*z*+2; (ii) *x*+1, *y*, *z*+1; (iii) −*x*+2, *y*+1/2, −*z*+5/2; (iv) *x*, −*y*+1/2, *z*−1/2; (v) −*x*+1, −*y*+1, −*z*+1; (vi) −*x*+1, *y*+1/2, −*z*+3/2; (vii) *x*−1, *y*, *z*−1; (viii) −*x*+1, −*y*, −*z*+1; (ix) −*x*+1, *y*−1/2, −*z*+3/2; (x) *x*−1, −*y*+1/2, *z*−3/2.
